# A Picture of the Present State of the Royal College of Physicians of London; Containing, Memoirs, Biographical, Critical, and Literary, of All the Resident Members of That Society; and of the Heads of the Medical Boards, with Some Other Distinguished Professional Characters : To Which Is Subjoined an Appendix, Containing an Account of the Different Medical Institutions of the Metropolis, Scientific and Charitable, with Their Present Establishments

**Published:** 1817-07

**Authors:** 


*A* PICTURE *of the present State of the* ROYAL COLLEGE *of* PHYSICIANS *of* LONDON; *containing, Memoirs, Biographical, Critical, and Literary, of all the Resident Members of that Society; and of the Heads of the Medical Boards, with some other distinguished Professional Characters: to which is subjoined an Appendix, containing an Account of the different Medical Institutions of the Metropolis, scientific and charitable, with their present Establishments.* London, 1817.

A MORE flimsy production we have seldom witnessed, if we except some few catch-penny works, evidently proceeding from the same manufactory. It consists of a few common-place remarks, and a scanty sprinkling of meagre anecdotes of various members of the College, and others. The very title page of the book, however, asserts a FALSITY on the face of it; *all* the resident physicians are not noticed, nor is any reason assigned for the selection: yet, we have been given to understand, that admittance was to be obtained by a golden key; but we think that man's vanity must be puerile indeed, which could be gratified by a niche in such a temple.

---

### PART III.

### SELECTIONS.

—— Factorum est copia nobis.

### FOREIGN JOURNALS.

### I. PHYSIOLOGY.—*Memoir on the nutritive Properties of Substances destitute of Azote.**

